# General anesthesia, using remimazolam, for the patient with myelin oligodendrocyte glycoprotein antibody associated disease (MOGAD): A case report

**DOI:** 10.1097/MD.0000000000031734

**Published:** 2022-11-18

**Authors:** Seung-Wan Hong, Byung-Soo Kim, Sang-Tae Park, Hae-Chang Jeong, Min-Sik Hwang, Seong-Hyop Kim

**Affiliations:** a Department of Anesthesiology and Pain Medicine, Konkuk University Medical Center, Konkuk University School of Medicine, Seoul, Korea; b Department of Infection and Immunology, Konkuk University School of Medicine, Seoul, Korea; c Department of Medicine, Institute of Biomedical Science and Technology, Konkuk University School of Medicine, Seoul, Korea.

**Keywords:** myelin oligodendrocyte glycoprotein antibody associated disease, remifentanil, remimazolam, total intravenous anesthesia

## Abstract

Myelin oligodendrocyte glycoprotein antibody associated disease (MOGAD) is one of auto-immune demyelinating diseases of nervous system. Although both regional anesthesia and general anesthesia has been successfully performed in the patient with demyelinating diseases of nervous system, it has been controversial which one is better.

**Patient concerns:**

Forty-four male patient was admitted for arthroscopic elbow surgery due to limitation of range of motion. The patient was diagnosed as MOGAD with anti-N-methyl-D-aspartate (NMDA) receptor encephalitis, and steroid was used to prevent and treat symptoms and signs.

**Diagnosis:**

He was diagnosed as MOGAD with anti-NMDA receptor encephalitis, 1 year ago. The patient complaint of dizziness, diplopia, nausea, vomiting, seizure, general weakness and so on when he was confirmed as MOGAD with anti-NMDA receptor encephalitis. The diagnosis of MOGAD was confirmed with positive anti-myelin oligodendrocyte glycoprotein (MOG) Immunoglobulin (Ig)G and negative anti-aquaporin 4 (AQP4) IgG in the blood.

**Interventions and outcomes:**

After steroid cover, total intravenous anesthesia (TIVA) with remimazolam and remifentanil was established for the patients. Rocuronium was administered under monitoring of neuromuscular blockade, using train of 4 (TOF). The operation was performed without any event under right lateral decubitus position. The patient was uneventfully recovered from anesthesia.

**Lessons:**

The case report showed total intravenous anesthesia with remimazolam and remifentanil under proper monitoring was successfully performed in the patient with MOGAD.

## 1. Introduction

Anesthetic management for the neurologic disease entities with demyelination in nervous system is always challenging. Although both regional anesthesia and general anesthesia has been successfully performed in the patient with it, it has been controversial which one is better.

Myelin oligodendrocyte glycoprotein antibody associated disease (MOGAD) is one of demyelinating diseases of nervous system. Although MOGAD is a different disease from multiple sclerosis (MS) but MOGAD has been occasionally misdiagnosed with MS.^[1–3]^ MOGAD is one of auto-immune disease. Both MDGAD itself, and the drugs for the prevention and the treatment of symptoms and signs should be considered to establish anesthesia plan for the patient with MOGAD.

We reported anesthetic management of the patient with MOGAD, undergoing arthroscopic elbow surgery. We successfully performed total intravenous anesthesia (TIVA) with remimazolam and remifentanil.

## 2. Case report

**Figure 1.**
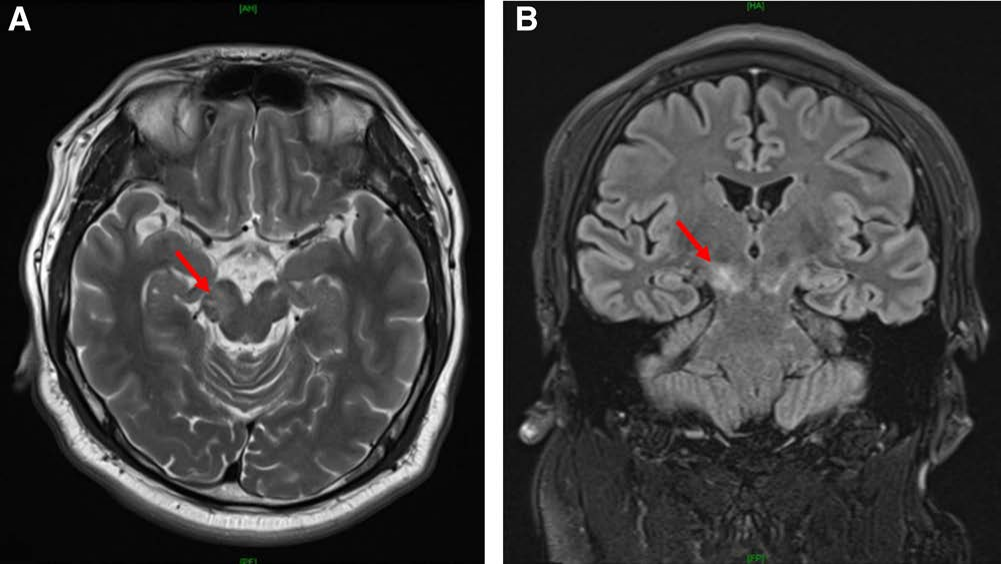
Brain magnetic resonance imaging of the right midbrain in T2 weighted image. (A) Axial image, (B) Coronary image. Red arrows showed ill-defined patchy hyperintense lesion in the right midbrain in T2 weighted image.

**Figure 2.**
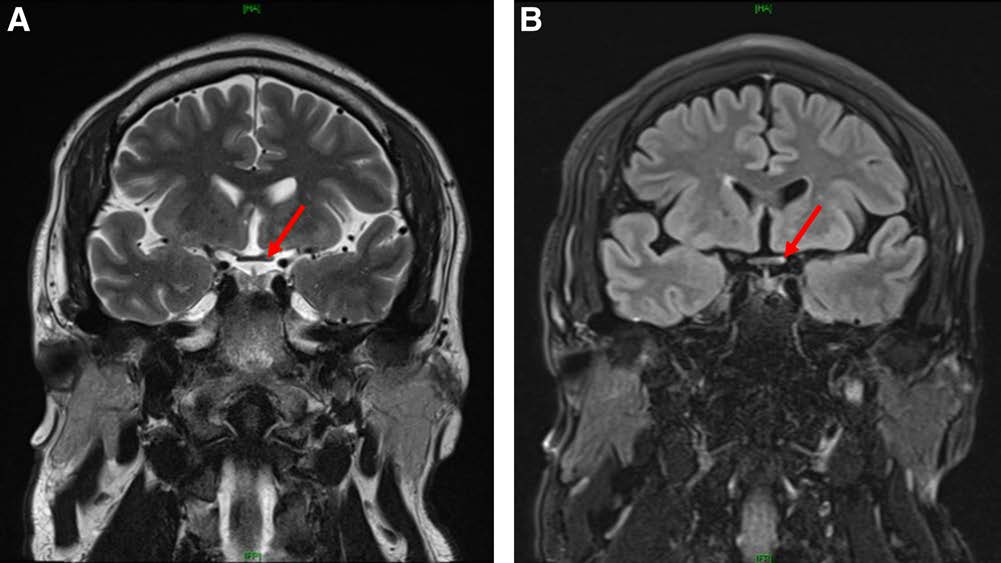
Brain magnetic resonance imaging of the left optic chiasm and the prechiasmatic optic nerve segment. (A) T2 weighted coronal image, (B) Fluid attenuated inversion recovery (FLAIR) image. Red arrows showed swelling with hyperintensity of the left optic chiasm and prechiasmatic optic nerve segment.

Written informed consent was obtained from the patient for publication of this report. Forty-four male patient with height, 172.5 cm and body weight 102.0 kg was admitted to perform arthroscopic elbow surgery due to limitation of range of motion. He was diagnosed as MOGAD with anti-N-methyl-D-aspartate (NMDA) receptor encephalitis, 1 year ago. The patient complaint of dizziness, diplopia, nausea, vomiting, seizure, general weakness and so on when he was confirmed as MOGAD with anti-NMDA receptor encephalitis. The diagnosis of MOGAD was confirmed with positive anti-myelin oligodendrocyte glycoprotein (MOG) Immunoglobulin (Ig)G and negative anti-aquaporin 4 (AQP4) IgG in the blood. Brain magnetic resonance imaging showed ill-defined patchy hyperintense lesion in the right midbrain in T2 weighted image (Fig. 1), and swelling with hyperintensity of the left optic chiasm and prechiasmatic optic nerve segment on T2 weighted image and fluid attenuated inversion recovery image (Fig. 2). After confirmation of MOGAD, steroid was used to manage it. He also had fatty liver, dyslipidemia, diabetes mellitus and avascular necrosis on both hips from the side effect of steroid. Present medication contained steroid (prednisolone 5 mg), immunosuppressive agent (mycophenolate mofetil), anticonvulsant [noncompetitive antagonist of α-amino-3-hydroxy-5-methyl-4-isoxazolepropionic acid receptor (perampanel) and lancosamide], lipid lowering agent (ezetimibe and rosuvastatin) and glucose lowering agent (dipeptidyl peptidase-4 inhibitor and biguanide) daily.

The patient was arrived at operating room without any premedication. Routine noninvasive monitorings (pulse oximeter, electrocardiograph, noninvasive blood pressure and bispectral index) were established. Hydrocortisone 50 mg was intravenously administered for steroid cover. Continuous infusion of insulin was set to control intraoperative glucose. Intravenous administration of remimazolam 0.15 mg/kg was followed by continuous infusion of remimazolam to maintain bispectral index level at 50 during anesthesia. After loss of consciousness and the confirmation of airway security, intravenous rocuronium 0.15 mg/kg was administered. Continuous infusion of remifentanil, using target-controlled infusion device, at 5.0 ng/mL at effect-site for Minto model,^[4]^ was started and maintained during anesthesia. Neuromuscular blockade was monitored, using train of 4 (TOF) at the right or left hand. After confirmation of TOF, 0, endotracheal intubation with endotracheal tube of inner diameter 8.0 mm was achieved, using video laryngoscope. The proper position of the endotracheal tube was confirmed by auscultation and ventilator was connected. Mechanical ventilation with fresh gas 2 L/m of fractional inspired oxygen 0.4, tidal volume 6 kg/mL and respiratory rate, adjusted by end-expiratory carbon dioxide at 40 mm Hg, and positive end-expiratory pressure 5 cmH_2_O.

The operation was performed without any event under right lateral decubitus position.

At the end of the operation, intravenous continuous infusions of remimazolam and remifentanil were stopped. Sugammadex 2 mg/kg was intravenously administered to reverse neuromuscular blockade. Extubation was performed after the confirmation for full recovery of neuromuscular blockade, using TOF. The patient was transferred into post-anesthetic care unit. Anesthesia time was 210 minutes and operation time was 165 minutes.

## 3. Discussion

MOGAD is a one of demyelinating disease of the nervous system. The incidence of MOGAD is rare but the patient with MOGAD has the chance to get the surgical intervention under anesthesia. Especially, the use of high dose steroid to suppress immune activation for the prevention and the treatment of MOGAD is associated with the need of orthopedic surgery.

MOGAD was considered as a variant of MS, because MOGAD shows demyelinating disease of nervous system, representing symptoms and signs, although MS does not show optic neuritis and MOGAD shows optic neuritis.^[5]^ However, MOGAD has the definite laboratory finding with positive MOG IgG, on the contrary to MS with negative auto-immune antibody.^[6]^ MOGAD is also differentiated from neuromyelitis optica spectrum disorder (NMOSD), one of auto-immune demyelinating disease of nervous system with optic neuritis, because MOGAD has negative AQP4 IgG but NMOSD had positive AQP4 IgG with negative MOG IgG (Table 1).^[7–9]^

**Table 1:** Characteristics of multiple scelorsis (MS), neuromyelitis optica spectrum disorder (NMOSD) and anti-myelin oligodendrocyte glycoprotein (MOG) associated disease (MOGAD).

		MS	NMOSD	MOGAD
Clinical manifestation		Discrete focal neurologic attacks including ON, TM, cerebellum and brainstem	ON, TM, or both; area postrema syndrome	ON, TM, or both; ADEM
Serologic antibody				
	AQP4 IgG	−	+	−
	MOG IgG	−	−	+

ADEM = acute disseminated encephalomyelitis, AQP4 = aquaporin 4, Ig = immunoglobulin, ON = optic neuritis, TM = transverse myelitis.

MOG is a glycoprotein and believed to play a role as a surface receptor or cell-adhesion molecule. The actual function of MOG had not been revealed yet. However, MOG has been thought to be an important role in myelination of nerves in central nervous system. MOG is located on the outermost lamellae and cell surface of oligodendrocyte, and a potential target from auto-immune antibody or demyelinated process involved cells.^[10,11]^

The patient also had anti-NMDA receptor encephalitis. It is also an auto-immune encephalitis and represent psychosis with seizure.^[12]^

The standard guideline for the treatment of MOGAD has not been established. However, intravenous steroid, intravenous immunoglobulin and exchange of plasma has been used to manage MOGAD because it is an auto-immune inflammatory demyelinating disease of nervous system.^[13]^

To establish anesthesia plan, 2 aspects should be concerned. MOGAD related problems and medication for MOGAD related problems. Both general anesthesia and regional anesthesia can be performed. However, there has been no study for the effect of anesthetic agents on MOGAD. Several report has shown the successful regional anesthesia in the patients with NMOSD, representing similar signs and symptom of MOGAD, except immunological findings. However, the results showed contradictory. There was a report with successful regional anesthesia in the patient with NMOSD.^[14]^ On the contrary, there was a report with aggravated symptoms and signs after several months, although successful regional anesthesia in the patient with NMOSD.^[15]^ When regional anesthesia is performed, the possibility that onset or offset of used local anesthetic agent is changed should be considered. The prolonged effect of used agents for general anesthesia should be cautious. Moreover, demyelinated nerve is susceptible to the toxicity of local anesthetic agent. If general anesthesia is chosen for the patient with MOGAD, neuromuscular blocking agent should be used and titrated under monitoring neuromuscular transmission.^[16]^ We performed TIVA with remimazolam and remifentanil, instead of inhalational anesthetic agent in the present case. Both intravenous anesthetic agent and inhalational anesthetic agent have been successfully used without suggestion of preference in demyelinating disease of nervous system. However, inhalational anesthetic agent usually increases intracerebral pressure (ICP) and we chose TIVA in the present case. Moreover, hepatic function should be cautious in the patient because he had fatty liver and took anticonvulsant, one of contributors for well-known liver toxicity. TIVA preserves hepatic blood flow than inhalational anesthetic agent. We did not use propofol, although propofol is well known to decrease ICP. On the aspect of ICP, propofol has the definite benefit. However, we concerned delayed emergence from general anesthesia due to MOGAD itself and decreased hepatic function, although propofol has the rapid context-sensitive half-time after intravenous continuous infusion. We used remimazolam, instead of propofol, with consideration for several characteristics of remimazolam. First, remimazolam has the rapid context-sensitive half-time, compatible with propofol. Second, remimazolam is rapidly metabolized by tissue esterase. Third, remimazolam has the specific anti-dote, flumazenil.

The patient took steroid to control MOGAD and arthroscopic elbow surgery is one of minor surgery. Therefore, intravenous hydrocortisone 50 mg before surgical incision was followed by intravenous hydrocortisone 25 mg at every 8 hours for 1 day. Aseptic procedure should be cautious because chronic medication of steroid with immunosuppressive agent and diabetes mellitus were the risk factors of infection, although the surgical procedure was minor. Therefore, glucose was strictly controlled by continuous intravenous infusion of insulin. MOGAD is accompanied by optic neuritis. The progression of MOGAD results in para- or quadri-plegia and is susceptible to pressure sore. Therefore, proper position during anesthesia to prevent eye damage or pressure sore should be cautious. Although anesthesia and surgical procedure were ended without any event, the stress from anesthesia and surgical procedure have the chance to progress or exacerbate the disease. Careful neurologic examination should be performed after anesthesia and surgical procedure.

In conclusion, the case report showed total intravenous anesthesia with remimazolam and remifentanil under proper monitoring was successfully performed in the patient with MOGAD.

## Author contributions

**Conceptualization:** Seung-Wan Hong, Seong-Hyop Kim.

**Investigation:** Seung-Wan Hong, Byung-Soo Kim, Sang-Tae Park, Hae-Chang Jeong, Min-Sik Hwang, Seong-Hyop Kim.

**Project administration:** Seong-Hyop Kim.

**Resources:** Seung-Wan Hong, Seong-Hyop Kim.

**Supervision:** Seong-Hyop Kim.

**Writing – original draft:** Seung-Wan Hong, Byung-Soo Kim, Sang-Tae Park, Hae-Chang Jeong, Min-Sik Hwang, Seong-Hyop Kim.
